# The determinants of stroke phenotypes were different from the predictors (CHADS_2 _and CHA_2_DS_2_-VASc) of stroke in patients with atrial fibrillation: a comprehensive approach

**DOI:** 10.1186/1471-2377-11-107

**Published:** 2011-08-24

**Authors:** Semi Oh, Suk Jae Kim, Soo-Kyoung Ryu, Gyeong-Moon Kim, Chin Sang Chung, Kwang Ho Lee, Oh Young Bang

**Affiliations:** 1 Department of Neurology, Stroke and Cerebrovascular Centre, Samsung Medical Centre, Sungkyunkwan University, South Korea

## Abstract

**Background:**

Atrial fibrillation (AF) is a leading cause of fatal ischemic stroke. It was recently reported that international normalized ratio (INR) levels were associated with infarct volumes. However, factors other than INR levels that affect stroke phenotypes are largely unknown. Therefore, we evaluated the determinants of stroke phenotypes (pattern and volume) among patients with AF who were not adequately anticoagulated.

**Methods:**

We analyzed data pertaining to consecutive AF patients admitted over a 6-year period with acute MCA territory infarcts. We divided the patients according to DWI (diffusion-weighted imaging) lesion volumes and patterns, and the relationship between stroke predictors (the CHADS_2 _and CHA_2_DS_2_-VASc score), systemic, and local factors and each stroke phenotype were then evaluated.

**Results:**

The stroke phenotypes varied among 231 patients (admission INR median 1.06, interquartile range (IQR) 1.00-1.14). Specifically, (1) the DWI lesion volumes ranged from 0.04-338.62 ml (median 11.86 ml; IQR, 3.07-44.20 ml) and (2) 46 patients had a territorial infarct pattern, 118 had a lobar/deep pattern and 67 had a small scattered pattern. Multivariate testing revealed that the CHADS_2 _and CHA_2_DS_2_-VASc score were not related to either stroke phenotype. Additionally, the prior use of antiplatelet agents was not related to the stroke phenotypes. Congestive heart failure and diastolic dysfunction were not associated with stroke phenotypes.

**Conclusions:**

The results of this study indicated that the determinants of stroke phenotypes were different from the predictors (i.e., CHADS2 and CHA_2_DS_2_-VASc score) of stroke in patients with AF.

## Background

Atrial fibrillation (AF) affects 3% to 5% of the population older than 65 years of age [1] and is a leading cause of fatal ischemic stroke [2,3]. Stroke in patients with AF is generally more severe and the outcome is markedly poorer than in patients with sinus rhythm [4]. Adjusted-dose warfarin is highly effective (~60% reduction), and aspirin is modestly effective (~20% reduction) for the prevention of stroke in AF [5,6]. Moreover, international normalized ratio (INR) levels at the time of stroke were recently reported to be associated with infarct volumes [7].

However, the factors affecting stroke phenotypes are unknown among AF patients. Several studies have suggested that infarct patterns are better delineated by DWI than other imaging modalities, are correlated with the underlying stroke pathogenic mechanism,[8,9] and are associated with stroke outcome [10]. In addition, the infarct pattern on DWI may differ depending on the characteristics (type and nature) of clots [11-13]. Risk factors for stroke (the CHADS_2 _and CHA_2_DS_2_-VASc score) and systemic (D-dimer and C-reactive protein) and local (left ventricular ejection fractions) factors may be associated with stroke phenotype. AF is associated with higher levels of prothrombotic factors, endothelial dysfunction, and markers of platelet activation [12,14,15]. The formation of left atrial appendage thrombi in AF is influenced by several hemodynamic factors that promote stasis, each of which can fluctuate over time, and perhaps also by mild prothrombotic hematological perturbations that favour thrombosis [6]. Therefore, we evaluated the determinants of stroke phenotypes (i.e., volume and pattern) among patients with AF who were not adequately anticoagulated (INR < 1.6) to identify factors besides the INR level that may influence the phenotypic presentation of stroke. The results presented here could lead to strategies for the prediction of stroke extent and improve clinical outcomes.

## Methods

### Patient selection

We analyzed demographic, clinical, laboratory, and radiographic data collected prospectively from consecutive patients admitted to Samsung Medical Centre for acute cerebral infarction from January 2005 through June 2011. Inclusion criteria for this study were: (a) presentation within three days of the onset of symptoms, (b) acute ischemic lesions within the MCA distribution on diffusion-weighted imaging (DWI), and (c) AF diagnosed previously or detected during the admission period. The exclusion criteria were: (a) patients who met the Trial of Org 10172 in the Acute Stroke Treatment algorithm competing diagnosis of large artery disease, small vessel, or other causes, (b) patients who did not have INR determination and DWI performed within 12 hours of initial presentation, and (c) patients who had INR levels greater than 1.6 (achieving INRs distributed equally in the range of 1.6 to 2.5 has been reported to provide 90% of the protection of INRs between 2.0 and 3.0 for primary prevention of stroke in patients with nonvalvular AF) [6]. The local Institutional Review Board approved the study and informed consent was obtained from participants.

### Work-up

All patients underwent MRI (3.0T, Achieva, Philips Medical Systems) using a protocol that also included DWI, gradient-recalled echo, fluid-attenuated inversion recovery, and magnetic resonance angiography (MRA) imaging of the cervical and intracranial vessels [16].

Variables that could potentially affect the acute infarct volume were recorded for each patient. These included sex, age, prior history of hypertension, diabetes, hyperlipidemia, valvular heart disease, coronary heart disease, time from symptom onset to MRI, and medications used before the onset of stroke including the statins, angiotensin receptor blockers, antiplatelet agent and warfarin. The CHADS_2 _score,[17] which is a simple method of estimating the risk of stroke in patients with non-rheumatic AF, was estimated from the clinical data and modified to correlate the score with stroke phenotype. Five risk factors are considered when determining the CHADS_2 _score: (a) C, recent congestive heart failure, (b) H, hypertension, (c) A, age ≥ 75 years, (d) D, diabetes mellitus, and (e) S, history of stroke or transient ischemic attack. In the present study, we used prior stroke or transient ischemic attack instead of the stroke index. In addition, we investigated use of the new CHA_2_DS_2_-VASc [18] score system for predicting the stroke phenotype, including additional stroke risk factors: (f) V, indicates vascular disease, (g) A, indicates age between 65-74 and (h) S, indicates sex category(female). The National Institutes of Health Stroke Scale (NIHSS) score on admission was also used to assess the severity of stroke. Stroke onset time was defined as the last time the patient was known to be free of symptoms. All patients underwent routine blood tests and high-sensitivity C-reactive protein (CRP) was taken as the impending cerebrovascular marker [19,20]. Hemostatic markers of prothrombotic tendency, including D-dimer and fibrinogen, were also evaluated. Patients underwent electrocardiography and cardiac telemetry for at least 24 hours to evaluate the persistence of AF. Paroxysmal AF is defined as intermittent periods of AF interposed with episodes of normal sinus rhythm, normally < 7 days [21]. In addition, echocardiogram was conducted in all but 12 patients who showed poor cooperation or acute illness owing to medical conditions or the stroke itself. During the echocardiogram, the size of the left atrium and left ventricular ejection fraction were measured,[22-25] and the E/e' ratio and DT were measured to diagnose the diastolic dysfunction [26].

DWI Infarct volume measurement: Methods and Image Analysis

DWI was obtained using two levels of diffusion sensitization (b values of 0 and 1000 s/mm^2^; 5-7 mm slice thickness; no gap). For each patient, the DWI lesion volumes were automatically outlined with subsequent manual corrections. The volumes were calculated using a computer-assisted volumetric analysis program (Medical Image Processing, Analysis and Visualization, Version 3.0, National Institutes of Health, Bethesda, Md). Raters outlined regions of acute diffusion abnormality on the b = 1000 s/mm^2 ^image while consulting the apparent diffusion coefficient and FLAIR sequences to distinguish acute from nonacute changes in diffusion. All acute DWI lesions were defined on a slice-by-slice basis using a semiautomatic threshold approach by a rater who was blinded to all clinical information.

### Infarct patterns

We divided the patients into three infarct topography pattern groups based on the observed DWI patterns: (a) territorial infarcts involving two or three MCA subdivisions (superior, inferior, or deep), (b) lobar infarcts involving one subdivision, large deep infarcts, and mixed cortical and deep infarcts, and (c) small (< 1 cm in diameter) scattered patterns, suggesting microembolism. Two readers blinded to the clinical data analyzed the DWI data, and the interobserver agreement (κ-value) was 0.92 (P < 0.001) for interpretation of the DWI lesion pattern (territory *vs*. lobar/deep *vs*. small scattered). The opinion of a third reader was obtained in cases of disagreement.

### Statistical analysis

All numeric data are presented as the mean ± SD or median (interquartile range [IQR]). The differences in DWI pattern and infarct volume and the clinical and laboratory parameters were evaluated by one-way ANOVA with post hoc analysis using the Fisher's least-square difference or Kruskall-Wallis test for continuous variables and Pearson's χ^2^, Fisher's exact test or linear by linear association for categorical variables. An independent t-test or the Mann-Whitney U test was used to evaluate differences in the factors between the groups. The agreement rate and κ-value between the infarct patterns reported by inter-observers was also obtained. Moreover, multivariate logistic regression analysis (stepwise method) was conducted to predict the independent contribution of factors that influenced stroke phenotypes (territorial pattern or highest quartile DWI lesion volumes). Variables from univariate analyses at P < 0.05 were considered to represent the explanatory variables and the other variables that we were specifically interested in; age, CHADS_2 _and CHA_2_DS_2_-VASc score and PT were entered for multivariate analysis. Among males in the present study, there were 29 current smokers and 24 ex smokers; however, there were only 2 female smokers and no female ex smokers. Accordingly, we did not analyze the impact of smoking on stroke phenotypes. P values < 0.05 were considered to be statistically significant.

## Results

Among 275 patients who met the inclusion criteria during the study period, only 27 (9.8%) had appropriate anticoagulation as indicated by an INR above 1.6 [6]. Finally, a total of 231 patients (118 men and 113 women; age, 70.0 ± 11.1 years) were included in this study. Reasons for exclusion of the remainder of the patients included probable other etiologies, the presence of large artery disease such as significant stenosis on ipsilateral carotid bifurcation (ten patients) and other causes (seven patients).

MRIs were completed within an average of 20.0 ± 28.9 hours of the event (range, 1-72 hours). The DWI lesion volumes ranged from 0.04-338.6 ml (median -11.86 ml; IQR, 3.07-44.20 ml). The clinical characteristics of the patients according to the DWI infarct volume quartile and infarct patterns are shown in Tables 1 and 2. NIHSS upon admission was associated with the subgroups of stroke phenotypes (P < 0.001). No correlation was observed between the levels of INR and DWI lesion volumes (r = -0.058, P = 0.495) (Additional file 1).

**Table 1:** Demographic and clinical characteristics among the infarct volume quartile groups

	Quartile of DWI lesion volumes, ml				*P*
		
	Q1 (< 3.1) n = 58	Q2 (3.1-11.8) n = 58	Q3 (11.9-44.2) n = 58	Q4 (> 44.2) n = 57	(Q4 vs. others)
NISS score on admission	4.0 ± 3.9	8.1 ± 6.1	10.8 ± 6.5	15.1 ± 6.8	< 0.001
Vascular risk factors					
Age	69.0 ± 10.9	70.0 ± 11.1	72.5 ± 10.4	69.5 ± 11.7	0.547
Male gender	24(41.4%)	26(44.8%)	32(55.2%)	36(63.2%)	0.036
Hypertension	41(70.7%)	41(70.7%)	42(72.4%)	36(63.2%)	0.250
Diabetes	10(17.2%)	13(22.4%)	7(12.1%)	14(24.6%)	0.222
Previous TIA/stroke	18(31.0%)	15(25.9%)	13(22.4%)	13(22.8%)	0.585
CHADS_2_*score					0.871
0-1	28(48.3.%)	24(41.4%)	25(43.1%)	25(43.9%)	
2-3	22(37.9%)	27(6.6%)	28(48.3%)	24(42.1%)	
4-5	8(13.8%)	7(12.1%)	5(8.6%)	8(14.0%)	
Dysrhythmia					
Paroxysmal atrial fibrillation	22(37.9%)	22(37.9%)	20(34.5%)	18(31.6%)	0.476
Concomitant valvular heart disease	5(8.8%)	9(15.5%)	5(8.6%)	5(8.9%)	0.663
Heart rate (/min)	78.1 ± 19.2	82.9 ± 21.6	85.1 ± 21.4	84.2 ± 23.2	0.480
Medications prior to onset					
Antiplatelet agents user	26(45.6%)	20(34.5%)	26(47.3%)	19(33.3%)	0.229
Warfarin user	7(12.1%)	9(15.5%)	4(6.9%)	8(14.0%)	0.610
Laboratory findings					
D-dimer, mg/dL	1.51 ± 2.85	2.01 ± 3.31	3.45 ± 12.87	2.06 ± 3.35	0.035
Fibrinogen, mg/dL	320.0 ± 83.9	303.0 ± 79.2	335.5 ± 81.9	346.6 ± 100.2	0.122
C-reactive protein, mg/dL	1.13 ± 2.95	0.69 ± 1.78	0.95 ± 2.04	0.80 ± 1.31	0.068
Prothrombin Time, INR	1.07 ± 0.11	1.10 ± 0.12	1.06 ± 0.11	1.06 ± 0.10	0.373
Transthoracic echocardiogram finding					
Left ventricular ejection fraction, %	62.1 ± 9.8	59.7 ± 12.4	60.2 ± 8.5	56.1 ± 15.4	0.020
Left atrium size, ml	48.0 ± 8.8	46.5 ± 9.4	47.8 ± 8.6	46.5 ± 7.2	0.660
E/e' ratio	13.20 ± 6.25	16.76 ± 9.85	13.84 ± 8.06	14.52 ± 9.00	0.839
DT, ms	230.5 ± 153.6	248.3 ± 154.5	207.3 ± 115.8	216.1 ± 114.2	0.925

*In CHADS_2_, S indicates prior stroke or transient ischemic attack

**Table 2:** Demographic and clinical characteristics among infarct pattern groups

	DWI lesion patterns			*P*
		
	Territory n = 46 (19.9%)	Lobar/Deep n = 118 (51.1%)	Small scattered n = 67 (29.0%)	(Territory vs. others)
NIHSS score on admission	16.3 ± 6.3	9.6 ± 6.1	4.1 ± 4.2	< 0.001
Vascular risk factors				
Age	68.3 ± .12.4	71.6 ± 11.4	69.3 ± 9.3	0.324
Male gender	29(63.0%)	57(48.3%)	32(47.8%)	0.070
Hypertension	28(60.9%)	84(71.2%)	48(71.6%)	0.733
Diabetes	10(21.7%)	20(16.9%)	14(20.9%)	0.603
Previous TIA/stroke	13(28.3%)	25(21.2%)	21(31.3%)	0.636
CHADS_2_*score				0.684
0-1	21(45.7%)	51(43.2%)	30(44.8%)	
2-3	18(39.1%)	55(46.6%)	28(41.8%)	
4-5	7(15.2%)	12(10.2%)	9(13.4%)	
Dysrhythmia				
Paroxysmal atrial fibrillation	15(32.6%)	44(37.3%)	23(34.3%)	0.647
Concomitant valvular heart disease	5(11.1%)	14(12.0%)	5(7.5%)	0.878
Heart rate (/min)	84.2 ± 25.1	84.2 ± 21.0	79.8 ± 19.9	0.501
Medications prior to onset				
Antiplatelet agents user	15(32.6%)	45(39.1%)	31(47.0%)	0.246
Warfarin user	8(17.4%)	12(10.2%)	8(11.9.%)	0.221
Laboratory findings				
D-dimer, mg/dL	2.37 ± 3.76	1.89 ± 3.19	1.29 ± 2.03	0.105
Fibrinogen, mg/dL	340.72 ± 100.6	324.5 ± 88.0	319.4 ± 77.7	0.230
C-reactive protein, mg/dL	0.84 ± 1.14	0.93 ± 2.22	0.87 ± 2.41	0.041
Prothrombin Time, INR	1.08 ± 0.11	1.07 ± 0.12	1.08 ± 0.10	0.667
Transthoracic echocardiogram finding				
Left ventricular ejection fraction, %	55.6 ± 16.3	60.0 ± 10.2	61.1 ± 11.1	0.008
Left atrium size, ml	47.2 ± 7.5	47.8 ± 9.2	46.4 ± 8.1	0.991
E/e' ratio	14.53 ± 10.21	15.11 ± 9.00	13.71. ± 6.16	0.478
DT, ms	237.4 ± 131.9	215.9 ± 118.9	244.8 ± 163.3	0.317

*In CHADS_2_, S indicates prior stroke or transient ischemic attack

**Figure 1:**
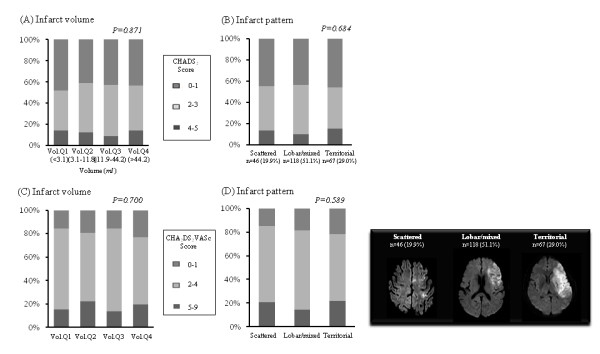
**The association of (A) infarct volume and (B) pattern with CHADS_2 _score and (C), (D) CHA_2_DS_2_-VASc score**.

The vascular risk factor profiles, the CHADS_2 _and CHA_2_DS_2_-VASc score, had no relationship with either stroke phenotype (Figure 1). Eighty-two patients (35.5%) had paroxysmal AF and the others had permanent AF. Twenty-eight patients were taking warfarin at sub-therapeutic levels, while 91 (39.3%) were taking antiplatelet agents. Interestingly, the prior use of medications including antiplatelet agents and warfarin was not related to the stroke phenotypes. We investigated the use of statin and angiotensin receptor blockers (ARBs), but data was not available for about 30% of the patients and the rest the proportion of the patients who had statins and ARBs was checked only 14.0%-24.1% and 18.5%-35.7%. Therefore, we could not evaluate the relationship between that medications and the stroke phenotype.

Compared with female, male gender was significantly associated with large infarct volumes (p = 0.036). Additionally, both systemic and local factors were associated with stroke phenotypes. Specifically, the lower left ventricular ejection fraction (p = 0.020) and higher D-dimer (p = 0.035) in patients was significantly associated with the highest quartile group of the DWI lesion volume, and the lower left ventricular ejection fraction was observed in patients with a territorial pattern (p = 0.008). Other variables showed no relationship with stroke phenotypes. Multiple logistic regression analysis was conducted to further evaluate the independent predictors for stroke phenotypes; however, systemic, local factors and CHADS_2 _and CHA_2_DS_2_-VASc score did not significantly add value of either stroke phenotype. (Additional file 2, Table S1 and S2).

## Discussion

Stroke affects not only the person who may be disabled, but their entire family and other caregivers [27]. In addition, more than half of the individuals at risk of stroke view a major stroke as being worse than death [27,28]. Identification of predictors of stroke phenotype and modification of these factors are particularly important in patients with AF. Despite recent advances in predicting the occurrence of AF-related stroke, little is known about the determinants of stroke phenotype in this type of ischemic stroke.

It was recently reported that the admission INR in preadmission warfarin users was inversely correlated with infarct volume as a result of the formation of a more fragile embolus, earlier recanalization or acceleration of thrombolysis [7]. In the present study, only 27 (9.8%) of 275 patients had appropriate anticoagulation as indicated by an INR above 1.6 [6]. Because of that small number and since we wanted to identify factors in addition to INR level that may influence the phenotypic presentation of stroke, we focused on patients who showed inadequate anticoagulation. The results of our study indicated that INR levels below the therapeutic range (< 1.6) were not correlated with infarct volumes, suggesting that only the therapeutic range of anticoagulation can modify the occurrence of stroke and phenotype in patients with AF [29]. In the present study, 91 (39.3%) patients were taking antiplatelet agents before admission. The use of antiplatelet agents before admission was also not related to the stroke phenotypes in the present study.

We next investigated the association of well-known risk factors for ischemic stroke and stroke phenotypes in patients with AF. Among the patients with AF, the absolute risk of stroke varies depending on patient age and other clinical features (associated stroke risk factors). The CHADS_2 _score, which is the clinical prediction rule for estimating the risk of stroke in patients with nonrheumatic AF, is the risk stratification scheme most widely used to predict AF-related thromboembolism [17]. In the present study, the CHADS_2 _and newly revised CHA_2_DS_2_-VASc score were not related to the stroke phenotype. Moreover, none of the CHADS_2 _components (e.g. hypertension and congestive heart failure) were associated with the stroke phenotype. Therefore, the results of the present study may indicate that the stroke phenotypes differed among patients with AF-related stroke, and that the factors related to the severity of stroke must be different from the known factors that contribute to the risk of stroke in AF.

Finally, local and systemic factors may also contribute to stroke phenotype in AF patients. The thrombi in AF patients have been strongly and consistently linked to stasis. Specifically, reduced blood flow velocities in the left atrial appendage (LAA) increase the duration of blood stasis in the LAA [6]. Recently, increased diastolic filling pressure has been found to be associated with a higher rate of left atrial thrombus in the AF, and these effects occur partially through blood stasis or impaired LAA function [30]. However, in the present study, the left atrial size, left ventricular dysfunction, diastolic dysfunction and the persistence of AF were not independently associated with stroke phenotypes. In addition to local factors (altered flow patterns in the LAA), hypercoagulable state, endothelial damage or inflammation, and platelet activation may all contribute to thrombus formation in AF [31-36]. In the present study, univariate analysis revealed that higher D-dimer (a degradation product of fibrinogen) levels were present in patients with territorial patterns. These findings reflect factors associated with thrombin-mediated fibrin formation and indicate that degradation of fibrinogen may be related to thrombus characteristics (size and fragility).

Strengths of this study included the consecutive recruitment of a well-phenotyped group of patients who underwent comprehensive workups, including various serological markers and echocardiogram. However, the results of this study should be interpreted with caution owing to the modest sample size and because all data were from a single centre. Moreover, endothelial activation and platelet activation were not assessed in this study. Additionally, TEE was only conducted in ten patients, while the other patients showed poor cooperation or acute illness due to medical conditions or the stroke itself.

## Conclusion

Our data showed that the determinants of stroke phenotypes were different from the predictors of stroke (i.e., CHADS_2 _and CHA_2_DS_2_-VASc risk scheme) in patients with AF who were inadequately anticoagulated. Further studies of different populations and larger cohorts are warranted.

## Competing interests

The authors declare that they have no competing interests.

## Authors' contributions

SO conceived of the study, performed the statistical analysis and drafted the manuscript. OYB conceived of the study, performed the statistical analysis and helped draft the manuscript. GMK, CSS and KHL participated in coordination and acquisition of data. SJK conduct data acquisition and statistical analysis. SR participated in the acquisition of data. All authors read and approved the final manuscript.

## Pre-publication history

The pre-publication history for this paper can be accessed here:

http://www.biomedcentral.com/1471-2377/11/107/prepub

## Supplementary Material

Additional file 1 **The association of infarct volume and international normalized ratio upon admission**. Click here for file

Additional file 2 **Table S1**. Logistic regression analysis for the highest quartile DWI lesion volumes and territorial infarct pattern group. Table S2. Logistic regression analysis for the highest quartile DWI lesion volumes and territorial infarct pattern group. (Including CHA2DS2-VASc score instead of CHADS2). Click here for file
