# Supplementary figures and images for: The determinants of stroke phenotypes were different from the predictors (CHADS2 and CHA2DS2-VASc) of stroke in patients with atrial fibrillation: a comprehensive approach
Source: BMC Neurol. 2011 Aug 24;11:107. doi: 10.1186/1471-2377-11-107 (PMC3174877; doi:10.1186/1471-2377-11-107)

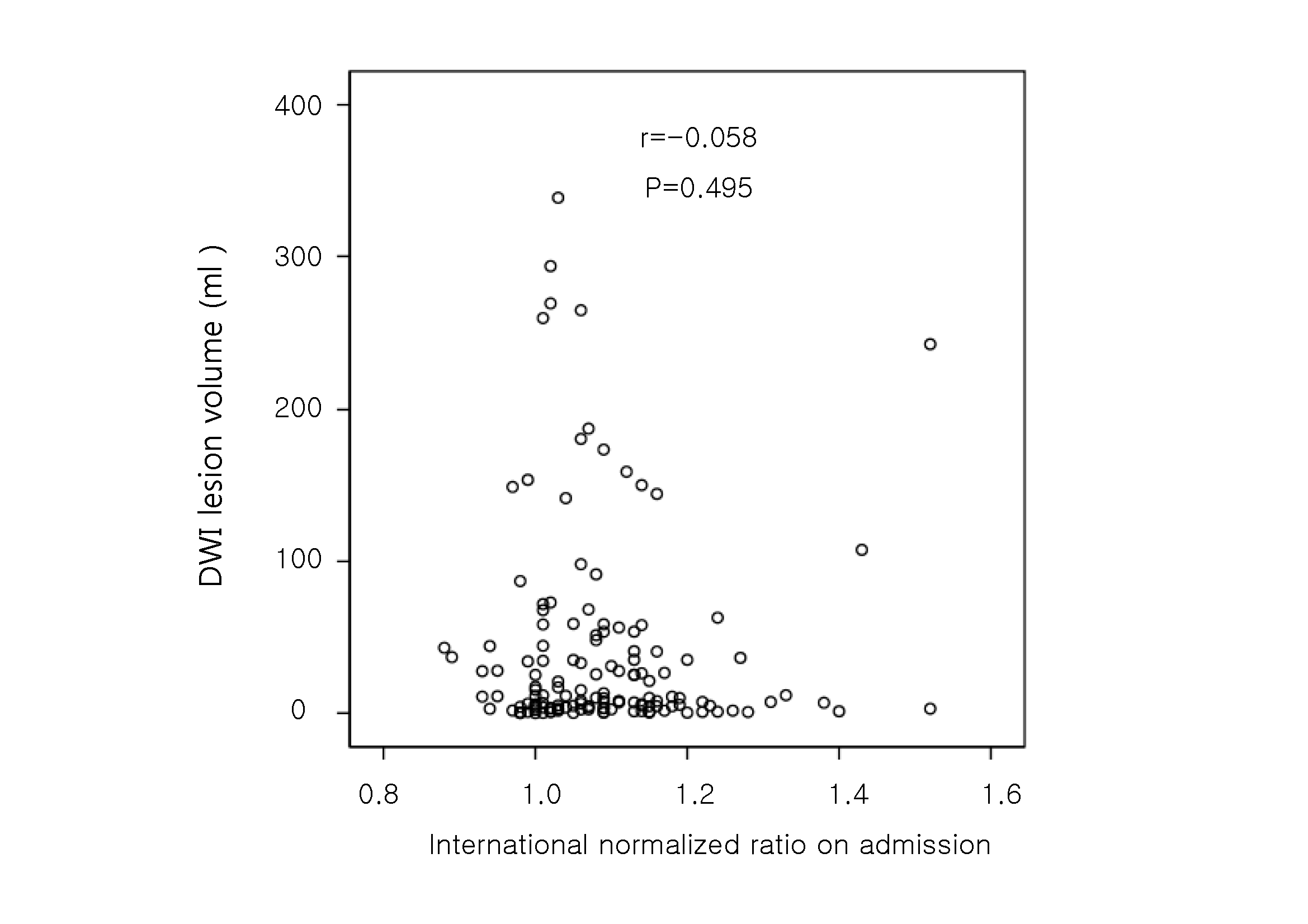

Supplement: Additional file 1 — The association of infarct volume and international normalized ratio upon admission. [file 1471-2377-11-107-S1.TIFF]
