# Supplementary material for: The determinants of stroke phenotypes were different from the predictors (CHADS2 and CHA2DS2-VASc) of stroke in patients with atrial fibrillation: a comprehensive approach
Source: BMC Neurol. 2011 Aug 24;11:107. doi: 10.1186/1471-2377-11-107 (PMC3174877; doi:10.1186/1471-2377-11-107)
Supplement: Additional file 2 — Table S1. Logistic regression analysis for the highest quartile DWI lesion volumes and territorial infarct pattern group. Table S2. Logistic regression analysis for the highest quartile DWI lesion volumes and territorial infarct pattern group. (Including CHA2DS2-VASc score instead of CHADS2). [file 1471-2377-11-107-S2.DOC]

Table S1. Logistic regression analysis for the highest quartile DWI lesion volumes and territorial infarct pattern group.

|  | Estimated odds ratio (OR) for  highest quartile DWI lesion volumes | | | | | |  | | Estimated odds ratio (OR) for  territorial infarct pattern | | | | |
| --- | --- | --- | --- | --- | --- | --- | --- | --- | --- | --- | --- | --- | --- |
|  | Crude |  | Multivaraite  OR (95%CI) | *P* | |  | | Crude | | |  | Multivaraite  OR (95%CI) | *P* |
| Vascular risk factors |  |  |  | |  |  | | | |  |  |  |  |
| Age | 0.974 |  | *0.984(0.947-1.022)* | | *0.392* | |  | | | *N/A* |  | *N/A* | *N/A* |
| Male gender | 1.946 |  | *1.716(0.817-3.604)* | | *0.154* | |  | | | 2.529 |  | *2.053(0.9274.543).* | *0.076* |
| CHADS_2_ score | 1.448 |  | *0.943(0.672-1.324)* | | *0.736* | |  | | | 1.746 |  | *0.838(0.610-1.151)* | *0.275* |
| Laboratory findings |  |  |  | |  | |  | | |  |  |  |  |
| D-dimer | 0.989 |  | *0.992(0.930-1.058)* | | *0.805* | |  | | | *N/A* |  | *N/A* | *N/A* |
| C-reactive protein | *N/A* |  | *N/A* | | *N/A* | |  | | | 0.977 |  | *0.991(0.823-1.193)* | *0.924* |
| Prothrombin Time | 0.389 |  | *0.419(0.016-10.830)* | | *0.600* | |  | | 0.870 | |  | *5.485(0.243-123.658)* | *0.284* |
| Transthoracic echocardiogram finding |  |  |  | |  | |  | |  | |  |  |  |
| Left ventricular ejection fraction | 0.986 |  | *0.983(0.951-1.015)* | | *0.295* | |  | | 0.969 | |  | *0.971(0.940-1.002)* | *0.065* |

Table S2. Logistic regression analysis for the highest quartile DWI lesion volumes and territorial infarct pattern group. (Including CHA_2_DS_2_-VASc score instead of CHADS_2_)

|  | Estimated odds ratio (OR) for  highest quartile DWI lesion volumes | | | | | |  | | Estimated odds ratio (OR) for  territorial infarct pattern | | | | |
| --- | --- | --- | --- | --- | --- | --- | --- | --- | --- | --- | --- | --- | --- |
|  | Crude |  | Multivaraite  OR (95%CI) | *P* | |  | | Crude | | |  | Multivaraite  OR (95%CI) | *P* |
| Vascular risk factors |  |  |  | |  |  | | | |  |  |  |  |
| Age | 0.974 |  | *0.988(0.945-1.032)* | | *0.581* | |  | | | *N/A* |  | *N/A* | *N/A* |
| Male gender | 1.946 |  | *1.581 (0.706-3.541)* | | *0.256* | |  | | | 2.529 |  | *1.843(0.715-4.750)* | *0.205* |
| CHA_2_DS_2_-VASc | 1.448 |  | *0.921(0.660-1.286)* | | *0.631* | |  | | | 1.746 |  | *0.7490.558-1.005)* | *0.054* |
| Laboratory findings |  |  |  | |  | |  | | |  |  |  |  |
| D-dimer | 0.989 |  | *0.992(0.930-1.058)* | | *0.811* | |  | | | 1.073 |  | *1.060(0.976-1.152)* | *0.163* |
| C-reactive protein | 0.929 |  | *N/A* | | *N/A* | |  | | | 0.977 |  | *0.968(0.767-1.221)* | *0.782* |
| Prothrombin Time | 0.389 |  | *0.445(0.017-11.673)* | | 0.627 | |  | | 0.870 | |  | *1.163(0.029-46.296)* | *0.205* |
| Transthoracic echocardiogram finding |  |  |  | |  | |  | |  | |  |  |  |
| Left ventricular ejection fraction | 0.986 |  | *0.92(0.950-1.015)* | | *0.266* | |  | | 0.969 | |  | *0.969(0.933-1.007)* | *0.112* |
